# Beyond the Peak – Tactile Temporal Discrimination Does Not Correlate with Individual Peak Frequencies in Somatosensory Cortex

**DOI:** 10.3389/fpsyg.2017.00421

**Published:** 2017-03-22

**Authors:** Thomas J. Baumgarten, Alfons Schnitzler, Joachim Lange

**Affiliations:** Institute of Clinical Neuroscience and Medical Psychology, Medical Faculty, University of DüsseldorfDüsseldorf, Germany

**Keywords:** beta, alpha, MEG, oscillations, perceptual cycles, temporal integration

## Abstract

The human sensory systems constantly receive input from different stimuli. Whether these stimuli are integrated into a coherent percept or segregated and perceived as separate events, is critically determined by the temporal distance of the stimuli. This temporal distance has prompted the concept of temporal integration windows or perceptual cycles. Although this concept has gained considerable support, the neuronal correlates are still discussed. Studies suggested that neuronal oscillations might provide a neuronal basis for such perceptual cycles, i.e., the cycle lengths of alpha oscillations in visual cortex and beta oscillations in somatosensory cortex might determine the length of perceptual cycles. Specifically, recent studies reported that the peak frequency (the frequency with the highest spectral power) of alpha oscillations in visual cortex correlates with subjects’ ability to discriminate two visual stimuli. In the present study, we investigated whether peak frequencies in somatosensory cortex might serve as the correlate of perceptual cycles in tactile discrimination. Despite several different approaches, we were unable to find a significant correlation between individual peak frequencies in the alpha- and beta-band and individual discrimination abilities. In addition, analysis of Bayes factor provided evidence that peak frequencies and discrimination thresholds are unrelated. The results suggest that perceptual cycles in the somatosensory domain are not necessarily to be found in the peak frequency, but in other frequencies. We argue that studies based solely on analysis of peak frequencies might thus miss relevant information.

## Introduction

The human sensory system is constantly excited by numerous stimuli originating from multiple sources. These stimuli often impinge on the sensory system within short time delays. Depending on the particular stimuli or situation, the sensory system needs to either integrate these stimuli into a temporally coherent percept or segregate these stimuli and treat them as temporally separate stimuli. Whether stimuli are perceived as temporally coherent or separated depends – among other factors – to a great part on the temporal distance between the stimuli. The role of temporal distance for perceptual integration has prompted the idea of ‘temporal integration windows,’ ‘perceptual cycles,’ or ‘perceptual moments’ (von Baer, 1908; Harter, 1967; VanRullen and Koch, 2003; VanRullen et al., 2014; Baumgarten et al., 2015; Cecere et al., 2015; Wutz et al., 2016). The concept of temporal integration windows or perceptual cycles states that the sensory system integrates input over a certain time window or cycle. Hence, stimuli falling within a certain time interval are perceptually integrated into one coherent percept. Vice versa, stimuli falling in two temporal windows are perceived as two distinct events. Although this concept is intriguing, computationally beneficial (Busch et al., 2009; Jensen et al., 2014) and has gained substantial evidence from behavioral studies (e.g., Sugita and Suzuki, 2003; Van Wassenhove et al., 2007), evidence for potential underlying neuronal mechanisms has been sparse.

One potential mechanism that has been repeatedly suggested as the neuronal concept of temporal integration windows are neuronal oscillations (VanRullen et al., 2014; Cecere et al., 2015; Landau et al., 2015). Several studies have shown that the phase of neuronal oscillations is linked to perception and behavior (Busch et al., 2009; Dugué et al., 2011; Landau et al., 2015; Gundlach et al., 2016). Phases of neuronal oscillations repeat periodically. Accordingly, several behavioral studies have shown that perception and behavior follow periodical and rhythmic patterns (Landau and Fries, 2012; Song et al., 2014; Huang et al., 2015). In addition, recent studies using EEG/MEG in humans have suggested that cycles of specific neuronal oscillations form the potential mechanism for temporal integration/segregation windows and correlate with perceptual reports. This could be shown, for example, in the visual cortex employing the wagon wheel illusion (VanRullen et al., 2006). In this paradigm, a wheel, although constantly rotating in one direction, is sometimes perceived as spontaneously reversing its direction of rotation. VanRullen et al. (2006) could show that the wagon wheel illusion correlates with cycles in the alpha (8–12 Hz) band oscillation in occipital areas. Furthermore, a study combining EEG and transcranial alternating current stimulation (tACS) provided causal evidence for alpha oscillations acting as temporal integration windows in an audio–visual illusion (Cecere et al., 2015). The study used the so-called double-flash illusion, where two auditory stimuli presented with one visual stimulus repeatedly induce the percept of a second, illusory visual stimulus if the three stimuli are presented with short temporal delays (typically < 100 ms; Shams et al., 2000). Cecere et al. (2015) showed that the individual temporal window for the audio–visual illusion correlated with the individual’s peak frequency of an alpha oscillation (i.e., those frequencies with the highest spectral power within the alpha-band) in parieto-occipital areas. More importantly, they showed that non-invasively manipulating the peak frequency and thus the length of the individual alpha cycles by means of tACS correlated with an increase or decrease, respectively, of the behavioral temporal integration windows.

In addition, a recent EEG study suggested that the peak frequency of parieto-occipital alpha oscillations might also represent a mechanism for temporal discrimination of visual stimuli (Samaha and Postle, 2015). The authors presented two visual stimuli separated by a blank gap or one visual stimulus with an identical overall temporal length, with subjects asked to report if they perceived stimulation as one single stimulus or two temporally separate stimuli. The authors showed that the individual length of the stimulus necessary for the respective subject to segregate two stimuli from one stimulus correlated with the subjects’ individual alpha peak frequency derived from occipital sensors.

Although the majority of studies on perceptual cycles focus on the visual domain, recent studies investigated mechanisms of temporal discrimination in the somatosensory domain. Baumgarten et al. (2015) used two electrotactile stimuli and determined neuronal correlates of the time windows perceptually separating the two presented stimuli. The study revealed that beta (13–20 Hz) and to a lesser degree also alpha (8–12 Hz) oscillations act as temporal integration windows (or perceptual cycles) in the somatosensory domain. This finding is consistent with the higher temporal resolution of touch compared to vision and the prominent role of beta band oscillations in the somatosensory domain (Jones et al., 2010; Haegens et al., 2011). In contrast to previous studies focusing on the visual domain (Cecere et al., 2015; Samaha and Postle, 2015), however, Baumgarten et al. (2015) did not explicitly analyze peak frequencies but phase differences between all frequencies from 5 to 40 Hz. Thus, it remains unclear whether the peak frequency of neuronal oscillations in the somatosensory domain also might act as a correlate for perceptual cycles.

In summary, recent studies provided novel evidence for the hypothesis that neuronal oscillations represent a putative neuronal mechanism for perceptual cycles in the visual and somatosensory domain. Studies on visual (Samaha and Postle, 2015) and audio–visual (Cecere et al., 2015) tasks suggest that the peak frequency of alpha oscillations in parieto-occipital areas represents the best estimate. However, it is unknown whether similar mechanisms hold true for the somatosensory domain, i.e., whether the peak frequency of the alpha- or beta-band is the best representation of the perceptual cycles in somatosensory regions. Similar to a study focusing on the visual domain (Samaha and Postle, 2015), the present study aimed to investigate this question by investigating whether somatosensory peak frequencies correlate with perceptual discrimination thresholds in a tactile temporal discrimination task. We hypothesized to find a negative correlation between individual discrimination thresholds and individual peak frequencies. That is, shorter discrimination thresholds should correlate with higher frequencies, i.e., shorter perceptual cycles/temporal integration windows.

## Materials and Methods

The subjects, experimental paradigm and MEG data investigated in the present study were previously reported in Baumgarten et al. (2015, 2016b). Here, we present a concise overview.

### Subjects

Sixteen right-handed volunteers [7 males, age: 26.1 ± 4.7 years (mean ± SD)] participated in the experiment after providing written informed consent in accordance with the Declaration of Helsinki and the Ethical Committee of the Medical Faculty, Heinrich Heine University Düsseldorf. Subjects reported normal or corrected-to-normal vision and no somatosensory and/or neurological disorders.

### Experimental Paradigm

**FIGURE 1:**
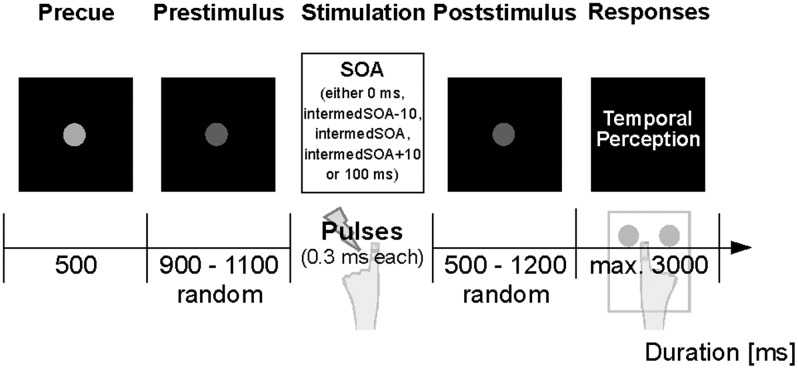
**Experimental paradigm.** The experiment started with a central fixation dot serving as start cue. A decrease in luminance after 500 ms signaled the start of the prestimulus epoch (jittered period of 900–1100 ms). Subsequently, electrotactile stimulation was applied to the left index finger with varying SOAs (0 ms, intermedSOA-10, intermedSOA, intermedSOA+10, 100 ms). Following a jittered poststimulus period (500–1200 ms), written instructions indicated the response window and subjects had to report their perception of the stimulation (perceived one stimulus vs. two stimuli) by button-press.

The present experimental paradigm is illustrated in **Figure 1** and described in Baumgarten et al. (2015, 2016b). Seated within the MEG, subjects were presented with electrotactile stimulation while visual instructions were projected on the backside of a translucent screen centrally positioned in front of the subjects. Trials began with a short precue period (500 ms; **Figure 1**), followed by a jittered prestimulus period (900–1100 ms). After the prestimulus period, either one or two electrical pulses were applied to the left index finger. Pulse amplitude was determined individually in a pre-measurement and set to a level above subjective perceptual threshold, but below pain threshold [4.1 ± 1.2 mA (mean ± SD)]. Pulses were separated by a specific stimulus onset asynchrony (SOA), which varied between 0 ms (i.e., only one pulse was presented) and 100 ms. Importantly, in the main condition subjects received pulses separated by an individually determined intermediate SOA [labeled *intermedSOA*; 25.9 ± 1.9 ms (mean ± SEM)] for which subjects reported a balanced perception of one stimulus or two stimuli (i.e., 50% of the trials were perceived as one stimulus, whereas the other 50% of the trials were perceived as two stimuli). In addition, two SOAs encompassed the intermedSOA by ± 10 ms (labeled *intermedSOA-10* and *intermedSOA*+*10*, respectively). Subsequent to stimulation, a jittered poststimulus period (500–1200 ms) was presented, after which subjects were indicated to report their respective perception (i.e., one or two stimuli) with a button press of the right hand. No feedback regarding the response was given.

### Psychometric Fitting Function

**FIGURE 2:**
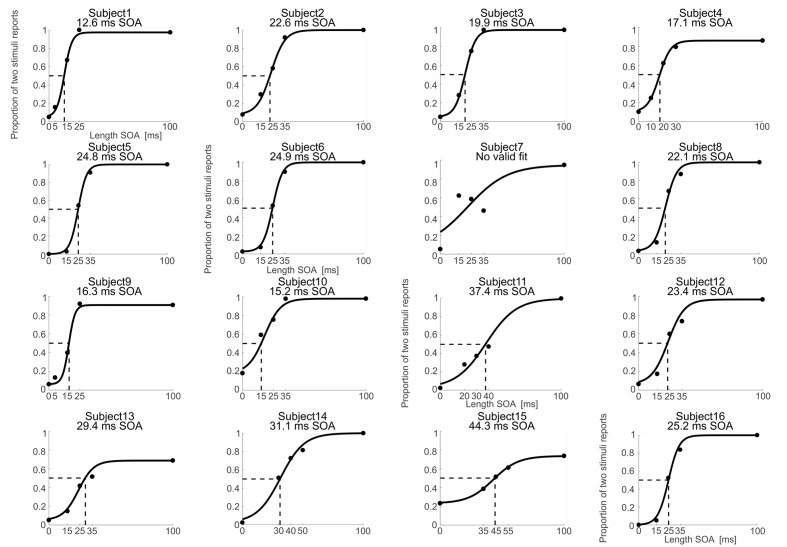
**Behavioral and fitting data.** Psychometric functions (black solid lines) were fitted to the individual proportion of ‘perceived two stimuli’ responses (black dots) as a function of the different SOAs. Black dotted lines indicate the SOA corresponding to a proportion of two stimuli reports of 0.5 (fitted intermedSOA). Exact fitted intermedSOAs for each subject are specified at the top of the individual figures. Note that for subject 7 the fit was not reliable so that subject 7 was excluded from further analyses.

The intermedSOA experimentally determined in the pre-experiment yielded naturally not an exactly equal distribution of perceived one and two stimuli in the main experiment, but some deviations. To determine the theoretical individual thresholds for which subjects achieve an equal distribution for the perception of one vs. two stimuli (the theoretical intermedSOA), we fitted psychometric functions to the experimental data of the main experiment (Cecere et al., 2015; Samaha and Postle, 2015). We fitted a sigmoid function to the data using the Palamedes toolbox for Matlab (Prins and Kingdom, 2009). The different experimental SOA lengths (i.e., 0 ms SOA, intermedSOA-10, intermedSOA, intermedSOA+10, 100 ms) were chosen as independent variable, whereas the individual proportion of ‘perceived two stimuli’ responses at each condition was chosen as dependent variable. The fit estimated four parameters: threshold, slope, guess rate, and lapse rate. Individual guess rates were set to the proportion of two stimuli percepts when actually one stimulus was presented (SOA 0 ms) and individual lapse rates to the proportion of one stimuli percepts when actually two stimuli with an SOA of 100 ms were presented. The goodness of fit was estimated by computing the deviance and corresponding *p*-values. *p*-values > 0.05 indicate a reliable fit of the experimental data. Only for one subject we found high deviance (*p* < 0.05), indicating that the data could not be reliably fitted (**Figure 2**, subject 7). This subject was excluded from further analyses. From the Palamedes toolbox, we determined thresholds at which subjects showed an equal distribution of perceiving one and two stimuli (**Figure 2**) and the corresponding error of the threshold.

### MEG Data Recording and Analysis

During the task, electromagnetic brain activity was continuously recorded by means of a 306-channel, whole-head MEG system (Neuromag Elekta Oy, Helsinki, Finland). Data was recorded with a sampling rate of 1 kHz. Only gradiometer data was analyzed for the present study. Since gradiometers are ordered in pairs of sensors measuring activity in mainly orthogonal directions, we offline combined these pairs of sensors to one sensor pair. This combination of orthogonal sensors resulted in 102 pairs of sensors. Offline analysis of the data was performed with custom-made MATLAB (Mathworks, Natick, MA, USA) scripts and the FieldTrip toolbox^1^ (Oostenveld et al., 2010).

Continuously recorded MEG data were segmented into trials, which started with the beginning of the precue period and ended with the subject’s response. Trials were visually and semi-automatically inspected for artifacts. Artifacts due to muscle activity, eye movements or technical reasons were removed semi-automatically by means of a *z*-score-based algorithm implemented in FieldTrip. Excessively noisy or dead channels were removed and reconstructed by an interpolation of neighboring channels. Power line noise was removed by applying a band-stop filter encompassing the 50, 100, and 150 Hz components. Furthermore, the linear trend and mean of every trial was removed from the data. Only trials with intermedSOA entered the subsequent analysis, which resulted in an average of 145 ± 19 trials with intermediate SOA (mean ± SD) after preprocessing.

We were interested in how the individual prestimulus alpha- (8–12 Hz) or beta- (14–30 Hz) band peak frequencies are related to the individual tactile temporal resolution. Thus, subsequent analyses focused on neuronal oscillations in sensorimotor areas during the prestimulus epoch of the respective trials (i.e., before any task- or response-related components). To analyze neuronal oscillations, data epochs from -900 to 0 ms relative to the onset of the first electrotactile stimuli were multiplied with a single hanning window, zero padded to a length of 10000 ms and fast Fourier transformed for frequencies from 5 to 40 Hz with a frequency resolution of 0.1 Hz. Gradiometer pairs were combined by summing spectral power across the two orthogonal channels, resulting in 102 channels.

**FIGURE 3:**
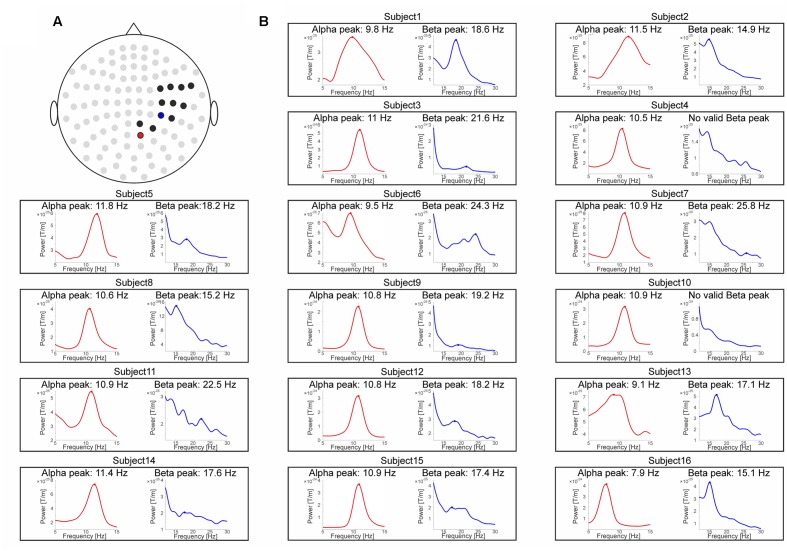
**MEG data.** **(A)** Sensor topography showing the sensors of interest (SOI) selection for the M50 ERF component (0.025–0.075 ms) as black dots. The single channel-pair showing maximum prestimulus (–900 to 0 ms) power levels on group level are marked as red dot for the alpha-band and as blue dot for the beta-band. **(B)** Spectral power representations for the alpha-band (red, left inset) and the beta-band (blue, right inset) for each subject. The respective peak frequencies are marked by a filled dot and specified above the subject-specific insets.

In order to focus the analysis on channels representing neural activity of the somatosensory cortex, the sensors of interest (SOI) were functionally determined by means of poststimulus event-related fields (ERFs) in response to electrotactile stimulation. ERFs were computed based on trials with intermedSOA. Trials were baseline corrected by subtracting the mean of the prestimulus period immediately preceding stimulus presentation (-200 to 0 ms). To focus on channels representing different components of activity from somatosensory cortex, we selected those time windows known to be critical for the different processing stages of somatosensory stimuli, i.e., the M50 of the ERF (known to origin mainly from S1) and M100 (known to originate mainly from S2; Suk et al., 1991; Iguchi et al., 2005). Therefore, amplitude values from 0.025 to 0.075 ms (labeled M50), 0.075–0.125 ms (labeled M100), and 0.025–0.125 (labeled M50+100) were averaged over all channels. Subsequently, those channels which amplitude values surpassed the respective average across all 102 channels by at least 1 SD were determined as the sensor-space for the respective somatosensory component (Supplementary Figure S1). The resulting channels included (MEG1122+23, MEG1132+33, MEG1312+13, MEG1322+23, MEG1332+33, MEG1342+43, MEG1442+43, MEG2022+23, MEG2222+23, MEG2232+33, MEG2242+43, MEG2412+13) for the M50 component (**Figure 3A**), (MEG0232+33, MEG1122+23, MEG1132+33, MEG1142+43, MEG1222+23, MEG1232+33, MEG1312+13, MEG1322+23 MEG1332+33, MEG1342+43, MEG1442+43, EG2212+13) for the M100 component and (MEG0232+33, MEG1122+23, MEG1132+33, MEG1142+43, MEG1222+23, MEG1312+13, MEG1322+23, MEG1332+33, MEG1342+43, MEG1442+43, EG2212+13) for the M50+100 component.

Similar to the approach Samaha and Postle (2015) chose for the visual domain, we defined two approaches to determine the individual peak frequencies. For the first approach, we selected the single channel-pair within the previously predetermined SOI showing on group level maximum prestimulus (-900 to 0 ms) alpha (8–12 Hz) or beta power (14–30 Hz), respectively. The rationale of this approach was that the channel-pair showing the maximum prestimulus power should provide the best estimate of peak frequency. Since the predetermined SOI slightly differed for the M50, M100, and M50+100 components, channels showing maximum power likewise differed across the respective components. The resulting maximum power channels for the beta-band were MEG2222+23 for the M50 component (**Figure 3A**), MEG0232+33 for the M100 component and MEG0232+33 for the M50+100 component. The resulting maximum power channels for alpha-band were MEG2022+23 for the M50 component (**Figure 3A**), MEG0232+33 for the M100 component and MEG0232+33 for the M50+100 component. Then we determined individual peak frequencies in these sensors (see below).

For the second approach, we determined the individual peak frequencies (see below) in the sensor showing on individual, single-subject level maximum prestimulus alpha or beta band power, again within the previously predetermined SOI. If no valid peak frequency could be found, peak frequency was determined in the sensor showing the second highest prestimulus power levels, and so on.

In addition to Samaha and Postle (2015), we also determined peak frequencies on source level by means of a “virtual sensor” approach. Here, we will give a concise description of the computation of virtual sensor data. For a detailed description of the procedure see Baumgarten et al. (2015). The virtual sensor was functionally determined by localizing the individual sources of the M50 or M100 component. Source localization was performed by means of an LCMV beamformer on individual 3D grids with a resolution of 1 cm. The grid points with maximal M50 or M100 activity were selected as the location of the virtual sensor (Supplementary Figure S2). In addition, we anatomically determined a virtual sensor for S1 based on the AFNI atlas implemented in FieldTrip, resulting in four neighbouring grid points (Supplementary Figure S2).

Next, we constructed spatial filters for the selected grid points. We projected single trial MEG sensor time series data through this spatial filter to obtain the time series data on source level. These time series data were then used as input to the frequency and peak detection analyses as described above. For the M50 and M100 defined virtual sensors, analysis was performed on single grid points, respectively. For the four atlas-defined virtual sensors, we performed spectral analysis separately for each sensor and then averaged spectral activity across the four grid points.

Individual alpha and beta peak frequency (IAFs and IBFs) were defined as the frequencies showing maximal power within the respective frequency band (8–12 Hz or 14–30 Hz). Peak frequencies were detected using the Matlab function findpeaks.m. In addition, to represent a peak, the power value of a potential peak frequency had to show an amplitude increase of at least 10% (i.e., MinPeakProminence was set to 10% of the amplitude of the peak). This method prevented peak frequency selection to be influenced by spontaneous power fluctuations and guaranteed that only peaks of sufficient size were selected as peak frequency.

**FIGURE 4:**
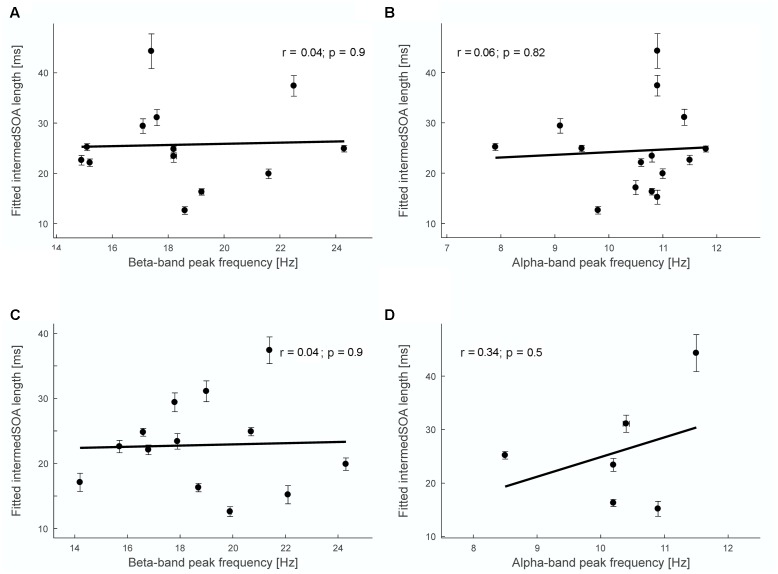
**Correlation analysis between fitted intermedSOA lengths and peak frequencies.** **(A)** Non-significant Pearson’s correlation between fitted intermedSOA lengths and beta-band peak frequency determined for sensor-level individual M50 sensors (*r* = 0.04, *p* = 0.9). **(B)** Non-significant Pearson’s correlation between fitted intermedSOA lengths and alpha-band peak frequency determined for sensor-level individual M50 sensors (*r* = 0.06, *p* = 0.82). **(C)** Non-significant Pearson’s correlation between fitted intermedSOA lengths and beta-band peak frequency determined for source-level M50 grid point (*r* = 0.04, *p* = 0.9). **(D)** Non-significant Pearson’s correlation between fitted intermedSOA lengths and alpha-band peak frequency determined for source-level individual M50 grid point (*r* = 0.34, *p* = 0.5). Insets in **(A–D)** show results of the linear regression analyses (black lines). Vertical arrow bars indicate standard errors of the fitted intermedSOA, horizontal error bars indicate the interquartile range for the bootstrap estimation of peak frequencies.

To obtain a measure of the reliability of the peak estimate, we performed a bootstrapping approach and recomputed the peak frequency 100 times. From this distribution of peak frequencies, we computed the interquartile range (**Figure 4**).

We additionally determined for each subject the theoretically expected frequency based on the models of perceptual cycles (Baumgarten et al., 2015, 2017; Samaha and Postle, 2015). According to these models, the cycle length of the theoretically relevant frequency should be determined by the intermedSOA:

$${Freq}_{theoretical}=1000/\left(2^{*}\mathrm{int}ermedSOA\right)$$

Finally, to test the hypothesis whether alpha- and beta-band frequencies are related (e.g., beta-band peak frequencies might be harmonics of the alpha-band peak frequencies), we investigated whether peak frequencies in the alpha- and beta-band are correlated by applying a Pearson correlation.

### Correlation Analysis and Bayes Factor Analysis

Correlations between IAFs and IBFs and individual theoretical intermedSOA were assessed by means of a Pearson correlation. The correlations were performed separately for each frequency band, SOIs and approach to determine the individual frequencies (group or single subject approach on sensor level or source level approaches).

In our study, we asked whether subjects’ temporal discrimination thresholds correlate with their individual peak frequencies. Using conventional inference statistics, however, it is only possible to provide evidence in favor of the H1-Hypotheses (i.e., correlation) by rejecting the H0-Hypotheses (no correlation). If the H0 cannot be rejected, this does not mean that the H0 (no correlation) is true. To test our two hypotheses of “no correlation” and “correlation” directly, we used Bayes factor (BF) analysis (Dienes, 2014; Iemi et al., 2016). In a nutshell, BF analysis tests whether the experimental data provide stronger evidence for the H0 or H1 hypothesis. A BF > 1 indicates more evidence for the H1, while BF < 1 indicates more evidence for H0. However, BF-values of 1/3–3 are regarded as inconclusive and only BF-values > 3 or <1/3 are regarded as providing sufficient evidence for H1 or H0. We computed the BF by forwarding the data of the correlation analysis to the BF analysis in the software JASP^2^.

## Results

### Behavioral Data (Temporal Resolution Thresholds)

In a pre-measurement, we determined the SOA for which subjects perceived two electrotactile stimuli as one stimulus in 50% of the trials, whereas in the other 50% of the trials the stimulation was perceived as two stimuli (labeled *intermedSOA*; **Figure 1**). In addition, trials with 0 ms SOA, intermedSOA-10, intermedSOA+10, 100 ms SOA were presented (see Materials and Methods for details).

On average, subjects perceived stimulation as two stimuli in 6.8 ± 1.5% (mean ± SEM) for trials with 0 ms SOA, in 25.8 ± 4.7% for trials with intermedSOA-10, in 58.0 ± 3.1% for trials with intermedSOA, in 79.4 ± 4.5% for trials with intermedSOA+10 and in 94.3 ± 2.4% for trials with 100 ms SOA.

Since in the main MEG experiment the intermedSOA did not yield a perfect equal distribution of “one” and “two” percepts, we fitted psychometric functions to the individual experimental data and computed the time point for which subjects theoretically perceived two successively presented stimuli as two stimuli in 50% of the trials and as one stimulus in 50% of the trials (**Figure 2**). The fitting procedure provided reliable fits for 15 out of the 16 subjects. The one subject showing a too high deviance (*p* < 0.05) and thus an unreliable fit was excluded from further analyses (subject 7, see **Figure 2**). The average intermedSOA across the 15 remaining subjects determined by the fitting procedure was 24.4 ± 2.2 ms (mean ± SEM; range 13–44 ms). We used these individual theoretically determined intermedSOA for the subsequent correlation analyses (see Correlations between IntermedSOAs and Peak Frequency and Bayes Factor Analyses).

### MEG Data (Peak Frequencies)

Individual alpha (8–12 Hz) and beta-band (14–30 Hz) peak frequencies were determined on sensor and on source level. On sensor level, we defined three functionally defined somatosensory SOI: M50, M100, M50+100 (see Materials and Methods for SOI definition and **Figure 3A** and Supplementary Figure S1 for illustration of the M50 SOI), in order to cover a wide range of potentially relevant sensors. We employed two different approaches for channel selection [i.e., group level analysis vs. single subject analysis (Samaha and Postle, 2015); see Materials and Methods for details].

For the single-subject analysis, valid beta-band peak frequencies could be determined in 13 out of the remaining 15 subjects for the M50 SOI and in all subjects in the M100 and M50+100 SOI. The average beta-band peak frequency was 18.5 ± 0.7 Hz (mean ± SEM) for the M50 SOI (see **Figure 3B** for individual spectra), 19.1 ± 0.8 Hz for the M100 channel selection and 19.1 ±0.8 Hz for the M50+100 SOI. Valid alpha peak frequencies could be determined in all subjects for the M50, M100, and M50+100 SOI. The average alpha-band peak frequency was 10.5 ± 0.3 Hz for the M50 SOI and 10.3 ± 0.2 Hz for the M100 and M50+100 SOI.

Visual inspection of the spectra confirmed the results of the automatic peak detection procedure for all reported peaks. In one case [M50 SOI, single-subject analysis of the beta-band (**Figure 3B**)], visual inspection might suggest an additional broad peak in one subject which was not detected by the automatic procedure. Including this subject based on peak definition by visual inspection, however, had only a negligible quantitative (absolute *r*-values became slightly smaller) and no qualitative effect on the correlation analysis. We will report the correlation analysis, however, only for the results of the objective peak detection procedure, thus excluding this single subject/peak from the respective correlation analysis.

For the group level analysis, valid beta-band peak frequencies could be determined in 9 out of 15 subjects for the M50 SOI and in 9 out of 15 subjects in the M100 and M50+100 SOI. The average beta-band peak frequency was 18.2 ± 0.6 Hz (mean ± SEM) for the M50 SOI and 18.6 ± 0.6 Hz for the M100 and M50+100 SOI. Valid alpha peak frequencies could be determined in all 15 subjects for the M50, M100, and M50+100 SOI. The average alpha-band peak frequency was 10.7 ± 0.2 Hz for the M50 SOI and 10.3 ± 0.2 Hz for the M100 and M50+100 SOI.

On source level, we defined regions of interest either based on the source localization of the M50 and M100 components or based on the AFNI atlas (see Materials and Methods for details).

Valid beta-band peak frequencies could be determined in 13 out of 15 subjects in the region defined by the M50 component, in 14 out of 15 subjects in the region defined by the M100 component and in 13 out of 15 subjects in the atlas-defined region. The average beta-band peak frequency was 18.9 ± 0.7 Hz (mean ± SEM) for the M50-defined region, 19.2 ± 0.9 Hz for the M100-defined region and 18.5 ± 0.7 for the atlas-defined region.

Valid alpha-band peak frequencies could be determined in 6 out of 15 subjects in each of the three regions. The average alpha-band peak frequency was 10.3 ± 0.3 Hz (mean ± SEM) for the M50-defined region, 10.9 ± 0.3 Hz for the M100-defined region and 10.1 ± 0.05 Hz for the atlas-defined region.

To test whether alpha- and beta-band peak frequencies are related (e.g., beta-peak frequencies being harmonics of the alpha-band peak frequencies), we performed a correlation analysis. None of the nine correlation analyses revealed a significant correlation between alpha- and beta-band frequencies (*r* < 0.27; *p* > 0.30).

### Correlations between IntermedSOAs and Peak Frequency and Bayes Factor Analyses

To determine any potential relationship between the temporal resolution of somatosensory perception and the alpha- or beta-band peak frequencies, we performed a correlation analysis between individual intermedSOAs from the fitting procedure [see Behavioral Data (Temporal Resolution Thresholds) and **Figure 2**] and the respective individual peak frequencies [MEG Data (Peak Frequencies) and **Figure 3B**].

**Figure 4** shows exemplary results for the correlation analyses. For the single subject analysis, no significant correlations were found on sensor level (M50 SOI) between intermedSOA and neither beta-band nor alpha-band peak frequencies (beta: *r* = 0.04, *p* = 0.90; alpha: *r* = 0.06, *p* = 0.82; **Figures 4A,B**). In addition, no significant correlations between intermedSOA and beta- or alpha-band peak frequencies were found on source level (M50 defined grid point; beta: *r* = 0.04, *p* = 0.90; alpha: *r* = 0.34, *p* = 0.5). The results of all correlation analyses are provided in detail in **Table 1**. In summary, none of the correlations revealed a significant correlation between intermedSOAs and peak frequencies. *r*-values varied between -0.19 and 0.35 (*p* > 0.22) for the beta-band and between -0.14 and 0.34 (*p* > 0.5) for the alpha-band, with *r*-values for the correlations on source level all being positive.

**Table 1:** Summary of the correlation analyses.

Frequency band of interest	Analysis on sensor or source level	Approach to define sensors of interest	No. subjects showing valid peak frequencies	*r*-value	*p*-value	Bayes-factor
Alpha	Sensor	Individual M50	15	0.06	0.82	0.27^∗^
	Sensor	Individual M100	15	0.08	0.77	0.26^∗^
	Sensor	Individual M150	15	0.08	0.77	0.26^∗^
	Sensor	Group M50	15	-0.14	0.61	0.49
	Sensor	Group M100	15	0.15	0.58	0.22^∗^
	Sensor	Group M150	15	0.15	0.58	0.22^∗^
	Source	M50	6	0.34	0.50	0.33^∗^
	Source	M100	6	0.33	0.52	0.34
	Source	Atlas	6	0.14	0.80	0.42
Beta	Sensor	Individual M50	13	0.04	0.90	0.31^∗^
	Sensor	Individual M100	15	-0.19	0.50	0.58
	Sensor	Individual M150	15	-0.19	0.50	0.58
	Sensor	Group M50	9	0.26	0.50	0.27^∗^
	Sensor	Group M100	9	0.02	0.96	0.39
	Sensor	Group M150	9	0.02	0.96	0.39
	Source	M50	13	0.04	0.90	0.31^∗^
	Source	M100	14	0.35	0.22	0.17^∗^
	Source	Atlas	12	0.19	0.55	0.24^∗^


*Peak frequencies were determined separately for the alpha- and beta-band (column 1) on either source or sensor level (column 2). The regions of interest in which peak frequencies were determined, were based on different approaches (column 3, see Materials and Methods for details). Column 4 shows for how many subjects peak frequencies could be determined. Results of the correlation analysis (*r*- and *p*-values are presented in columns 5 and 6. Finally column 7 shows the result of the Bayes factor analyses for each correlation. ^∗^Indicates strong evidence in favour of the null hypothesis of no correlation (i.e., BF < 1/3).*

**Table 1** also provides the results of BF analysis. In summary, the BF analysis revealed that for all correlations BF-values were <1, thus providing stronger evidence for the H0 hypothesis (i.e., there is no correlation) than for the H1 (i.e., there is a correlation). For the beta-band on sensor level, 2 out of 6 analyses provided strong evidence in favor of the H0 (BF < 1/3), while on source level, all three analyses provided strong evidence for the H0 (BF < 1/3, i.e., no correlation). For the alpha-band on sensor-level, 5 out of 6 analyses provided strong evidence for the H0, while on source level for one analysis BF was <1/3 while the other two BF-values were still ≤0.42 (note that on source level alpha peaks could be detected only for a small number of subjects).

Finally, to test whether experimentally and theoretically determined frequencies (see Materials and Methods and arrows in Supplementary Figure S3) are related to each other, we performed a correlation analysis. None of the correlations revealed a significant correlation (*r* < 0.27; *p* > 0.31).

## Discussion

It has long been debated whether perception is organized as a continuous process or in discrete perceptual cycles (or temporal integration windows), where two stimuli falling within one cycle are perceptually integrated to one stimulus and two stimuli falling in two separate cycles are perceived as two separate stimuli (von Baer, 1908; Harter, 1967; Allport, 1968; VanRullen and Koch, 2003). Recent studies have suggested that the peak frequency (i.e., the frequency with the maximal power) of alpha-band (8–12 Hz) oscillations in parieto-occipital areas serves as the neuronal correlate of such perceptual cycles in the (audio-) visual domain (Cecere et al., 2015; Samaha and Postle, 2015). Here, we studied in a tactile temporal discrimination task whether peak frequencies might likewise serve as a correlate for perceptual cycles in the somatosensory domain. However, we were unable to demonstrate a significant correlation between subjects’ individual peak frequencies and their perceptual temporal discrimination thresholds. This lack of correlation was true for the alpha- (8–12 Hz) and the beta- (14–30 Hz) band over several regions of interest in the somatosensory cortex on sensor level as well as on source level. Since from a lack of significant correlation it does not necessarily follow that the null hypothesis (i.e., there is actually no correlation) is true, we additionally performed an analysis of BF. The BF analysis revealed that for all tested correlations evidence was stronger in favor of the H0 (no correlation) compared to the H1 (correlation) as indicated in BF-values < 1. Importantly, most of the BF provided strong evidence for the H0 (BF-values < 1/3), especially those on source level.

We have performed our peak frequency detections on sensor and on source level. The reason to perform the analysis on sensor level was to keep the analyses as close as possible to previous studies which have performed their analyses on sensor level as well (Samaha and Postle, 2015; Cecere et al., 2015). This way, we ought to ensure that methodological approaches are similar, facilitating the comparability across studies. A disadvantage of the analysis on sensor level is the problem of spatial smearing. That is, sensors do not only measure activity from the region directly below them, but easily can pick up activity from more distant areas. For example, sensors over somatosensory areas might not only measure somatosensory alpha activity but also pick up alpha activity from parieto-occipital regions. Such activity might potentially deteriorate the analysis. Our source level analyses support this concern. While we found clear alpha peaks for all subjects on sensor level, only a minority of subjects showed alpha peaks on source level. These different results for sensor and source level analyses suggest that sensor and source data contain different signals with sensor level analysis being more prone to potential parieto-occipital alpha-band activity. Thus, we believe that while sensor level analyses ensure better comparability to previous studies, source level analyses are closer related to the actual somatosensory neuronal activity. Importantly, the correlation and BF analyses on source level demonstrated stronger evidence in favor of the H0 of no correlation (BF < 0.31 for all beta-band analyses; BF < 0.42 for all data analyses, please note the overall low number of subjects for alpha-band analyses), while the sensor level provided evidence for H0 but BF values were mostly in the “inconclusive” range (1/3 < BF < 3).

Importantly, it should be noted that this non-significant result does not imply that in the somatosensory system perceptual cycles do not exist. Rather, a previous study has shown that the phase of neuronal oscillations in the primary somatosensory cortex in the alpha-/beta- (8–20 Hz) band correlates with subjects’ perception, in line with the idea of perceptual cycles in the somatosensory domain (Baumgarten et al., 2015). Thus, while there is evidence for perceptual cycles, the present results state that the carrying frequency of the perceptual cycles in somatosensory areas is not necessarily equivalent to the peak frequency of a frequency band.

On the other hand, studies in the visual domain reported a significant correlation between alpha peak frequencies and perception or discrimination performance (Cecere et al., 2015; Samaha and Postle, 2015). This raises the question where these discrepancies between results in the visual and somatosensory domain originate from? One reason might be found in methodological differences between studies or in inapt parameters for the analyses. For example, too low statistical power due to a low number of subjects might account for a non-significant result. Our hypothesis was to find a negative correlation of individual perceptual thresholds and individual peak frequencies. That is, shorter thresholds should result in higher frequencies, i.e., shorter cycles. While we found a small, but non-significant negative correlation in a few correlations, it is unlikely that the correlation becomes significant with a higher number of subjects. This is mainly due to the reason that BF values provided stronger evidence for the null hypothesis of no correlation. While for a few regions of interest in which we analyzed spectral activity, BF-values are strictly speaking inconclusive (1/3 < BF < 3), other regions of interest show strong evidence in favor of the “no correlation” hypothesis. This is mostly evident for the correlation analyses on source level. As discussed above, we argue that source level analyses of peak frequencies should be more reliable than sensor level analyses. In addition, subjects showed a rather high variability with no obvious and consistent relationship between perceptual thresholds and individual peak frequencies. Also, we did not find a significant correlation between the experimentally determined peak frequencies and the relevant frequencies predicted by the models of perceptual cycles (Baumgarten et al., 2015, 2017; Samaha and Postle, 2015). Moreover, for most subjects, no clear peak could be detected at frequencies predicted by the model. This finding is difficult to explain by low statistical power alone. Moreover, the number of subjects in our study is comparable to the number of subjects in other studies that found a significant correlation in visual areas for alpha peak frequencies (Samaha and Postle, 2015). Secondly, we cannot exclude, of course, that with different parameters or a more fine-grained analysis, the hypothesized negative correlation can be found. However, we specifically chose our parameters to cover different regions and frequency bands of interest and different approaches to analyze peak frequencies (e.g., based on individual or group level). Furthermore, we tried to keep our analyses as close as possible to analyses of a previous study that reported to succeed in finding a significant result (Samaha and Postle, 2015). There are some differences, however, that might explain at least to some degree differences in the results. While we analyzed subjects’ perception of two short stimuli directly (i.e., if subjects perceived two stimuli separated by a specific SOA as one single stimulus or two separate stimuli), Samaha and Postle (2015) analyzed subjects’ ability to discriminate two rather long stimuli separated by a temporal gap from a single stimulus matching the duration of both stimuli and the respective temporal gap. It might be that this paradigm measures subjects’ ability to detect the gap between the two stimuli to a certain degree. However, this is only indirectly related to the perception of two discrete stimuli. Furthermore, the length of the stimuli differs considerably between both studies (i.e., a flash in the study from Samaha and Postle lasted 40 ms, whereas an electrotactile pulse in the present study lasted 0.3 ms). The to-be-expected differences in stimulus processing might therefore further hamper a comparison of the results. In another study that found a significant correlation between individual peak frequencies and temporal integration windows, Cecere et al. (2015) used a paradigm in which auditory and visual stimuli need to be integrated to induce a visual illusion. Their paradigm might thus focus stronger on the integration of crossmodal stimuli while our study focuses on the temporal segregation of unimodal stimuli. Such integration processes across sensory modalities might correlate more strongly with the peak frequencies in the alpha-band. Finally, definition of regions of interest differed slightly between studies. For example, while sensors were chosen in regions expected to be most directly related to prestimulus or stimulation effects in our study and by Cecere et al. (2015), Samaha and Postle (2015) chose SOI in a wider spatial range, potentially covering some sensors not directly related to stimulus processing (e.g., potentially ipsilateral to processing sites). However, while these methodological differences might explain some differences of the results, they cannot fully explain the lack of significant correlations in our study. This is mainly due to the fact that the methodological differences also partly exist between Cecere et al. (2015) and Samaha and Postle (2015), yet both studies found significant correlations in the visual domain.

One simple argument to explain the differences might be that neuronal oscillations in visual and somatosensory regions have different characteristics. While alpha-band activity is mostly characterized by one strong and more or less clearly defined peak, beta-band activity is sometimes characterized by multiple, weaker peaks (see **Figure 3B**) or even no clear peak, at all. In line with these results, we did not find a significant correlation between alpha and beta band peak frequencies. Thus, we feel safe to exclude that beta-band peak frequencies might be simply harmonics of the alpha-band activity (see also Haegens et al., 2014). In addition, we found that some subjects did not show a reliably detectable peak in the alpha band on source level, although a peak in the beta-band could be reliably determined. Thus, it might be that not the peak with the highest power is the carrier frequency of perceptual cycles, but other peaks with overall lower power. Additionally, it should be noted that although several effects and/or functions seem to be reflected in the peak frequency (Salmelin and Hari, 1994; Haegens et al., 2011; Baumgarten et al., 2016a), functionally significant effects do not need to be necessarily reflected in the peak frequency (e.g., Pavlidou et al., 2014; Tucciarelli et al., 2015). Thus, it might well be that the carrier frequency is not reflected in any peak, e.g., potentially because the carrier frequency is characterized more strongly by phase rather than power (Baumgarten et al., 2015).

Despite the lack of a significant correlation between peak frequencies and perceptual performances in our study in the somatosensory domain and thus the discrepancy to other recent studies in the visual domain (Cecere et al., 2015; Samaha and Postle, 2015), there is increasing evidence for neuronal oscillations as the correlate of discrete perceptual cycles (VanRullen et al., 2014; Baumgarten et al., 2015). The studies arguing in favor of perceptual cycles commonly agree on the hypothesis that the length of a cycle of a neuronal oscillation determines a perceptual cycle for integration or segregation. The discrepancy seems to be which frequency determines the relevant oscillation and how the frequency can be determined. We suggest that the phase of a neuronal oscillation is a critical measure that determines the perceptual cycle (Baumgarten et al., 2015). The critical role of phase is supported by recent studies which reported a correlation between the phase of neuronal oscillations and perception or behavior (Busch et al., 2009; Mathewson et al., 2009; Busch and VanRullen, 2010; Dugué et al., 2011; Schyns et al., 2011; VanRullen et al., 2011; Landau and Fries, 2012; Landau et al., 2015). The phase might determine the beginning and end of a perceptual cycle. The phase and the power of an oscillation might carry different information and thus act independently or in different frequencies (Schyns et al., 2011). Thus, effects of phase might be independent of power and thus might be found in frequencies that do not show the maximal power (e.g., Baumgarten et al., 2015). On the other hand, phase and power effects might be found in the same frequency, especially if there is only one frequency active in a certain neuronal system (e.g., presumably the alpha oscillations in the visual system). We thus suggest that analyzing the power or peak frequency is a relevant tool for determining perceptual cycles and their carrier frequency. This approach might, however, sometimes miss relevant information which is coded in the phase of an oscillation. This might be an explanation why we found evidence for perceptual cycles when analyzing the phase of beta oscillations in the somatosensory domain (Baumgarten et al., 2015) but not in the present study when analyzing the peak frequencies. We thus suggest broadening analyses of spectral power to phases in broader frequency bands rather than focusing purely on peak frequencies.

Future studies might also use more strongly modulatory techniques to establish a causal link between putative perceptual cycles, oscillations and perception. tACS might be used to non-invasively modulate the length of perceptual cycles (Cecere et al., 2015). In addition, studies have demonstrated that beta oscillations can be pharmacologically modulated (Hall et al., 2011; Muthukumaraswamy et al., 2013). Such modulations might be used to modulate beta oscillations in somatosensory cortex and measure the effect on putative perceptual cycles and consequently perception.

## Conclusion

There is cumulative evidence for perceptual cycles in visual and somatosensory cortex resulting in discrete and cyclic perception. While some studies on the visual domain argue that the peak frequency acts as the neuronal correlate of perceptual cycles, we were unable to demonstrate such a correlation between peak frequencies and perception in the somatosensory domain in the present study. We argue that this discrepancy does not speak against perceptual cycles, at all, but for an analysis that goes beyond analyses of power and peak frequencies, taking the phase of neuronal oscillations into account.

## Author Contributions

JL conceived the study, TB and JL designed the study, TB collected the data, TB and JL analyzed the data, TB, AS, and JL wrote the article.

## Conflict of Interest Statement

The authors declare that the research was conducted in the absence of any commercial or financial relationships that could be construed as a potential conflict of interest.

## Footnotes

1 fieldtriptoolbox.org

2 https://jasp-stats.org, version 0.8.0.1
