# Supplementary figures and images for: Beyond the Peak – Tactile Temporal Discrimination Does Not Correlate with Individual Peak Frequencies in Somatosensory Cortex
Source: Front Psychol. 2017 Mar 22;8:421. doi: 10.3389/fpsyg.2017.00421 (PMC5361566; doi:10.3389/fpsyg.2017.00421)

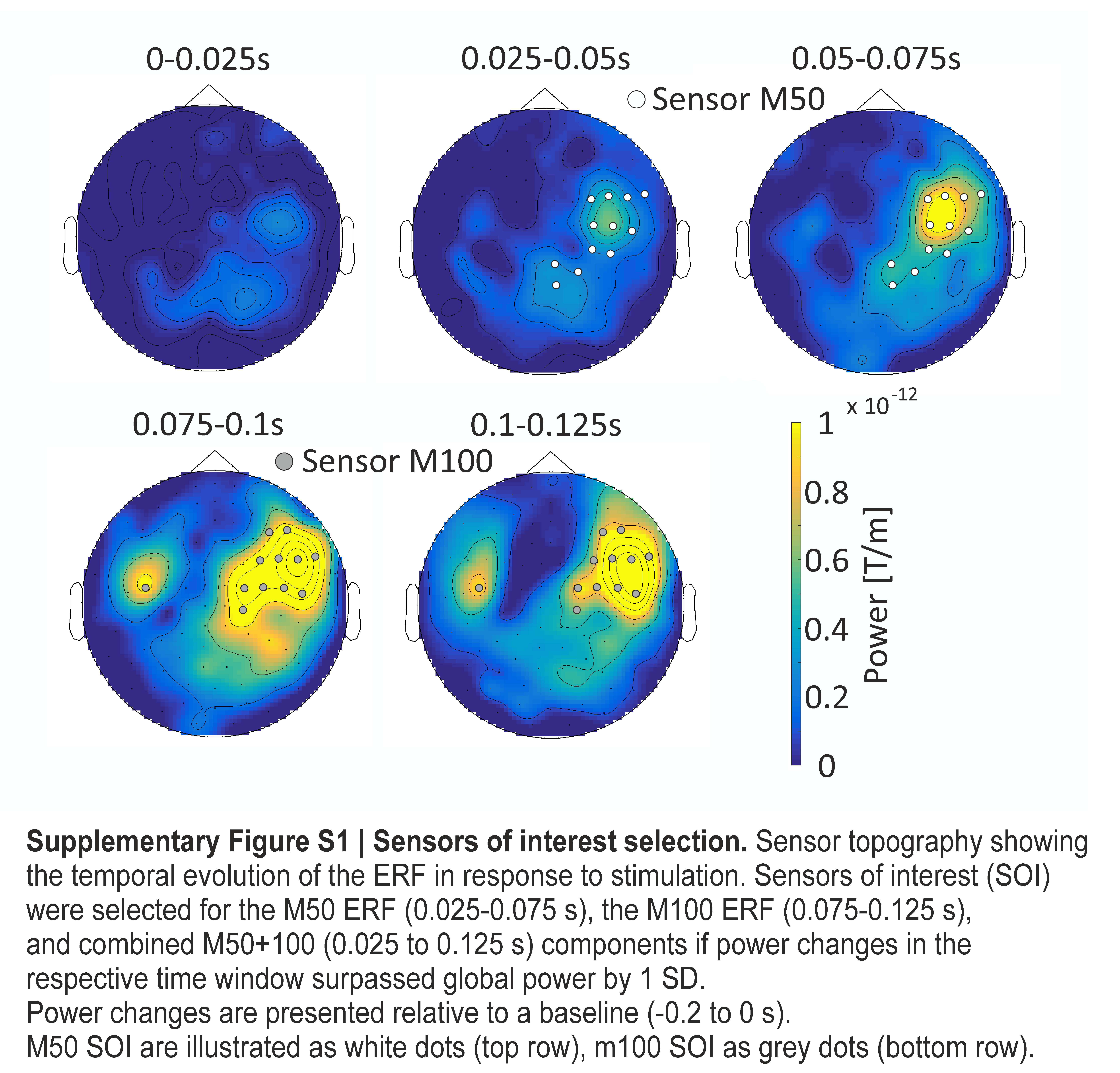

Supplement: Supplementary file 1 [file Image_1.tif]

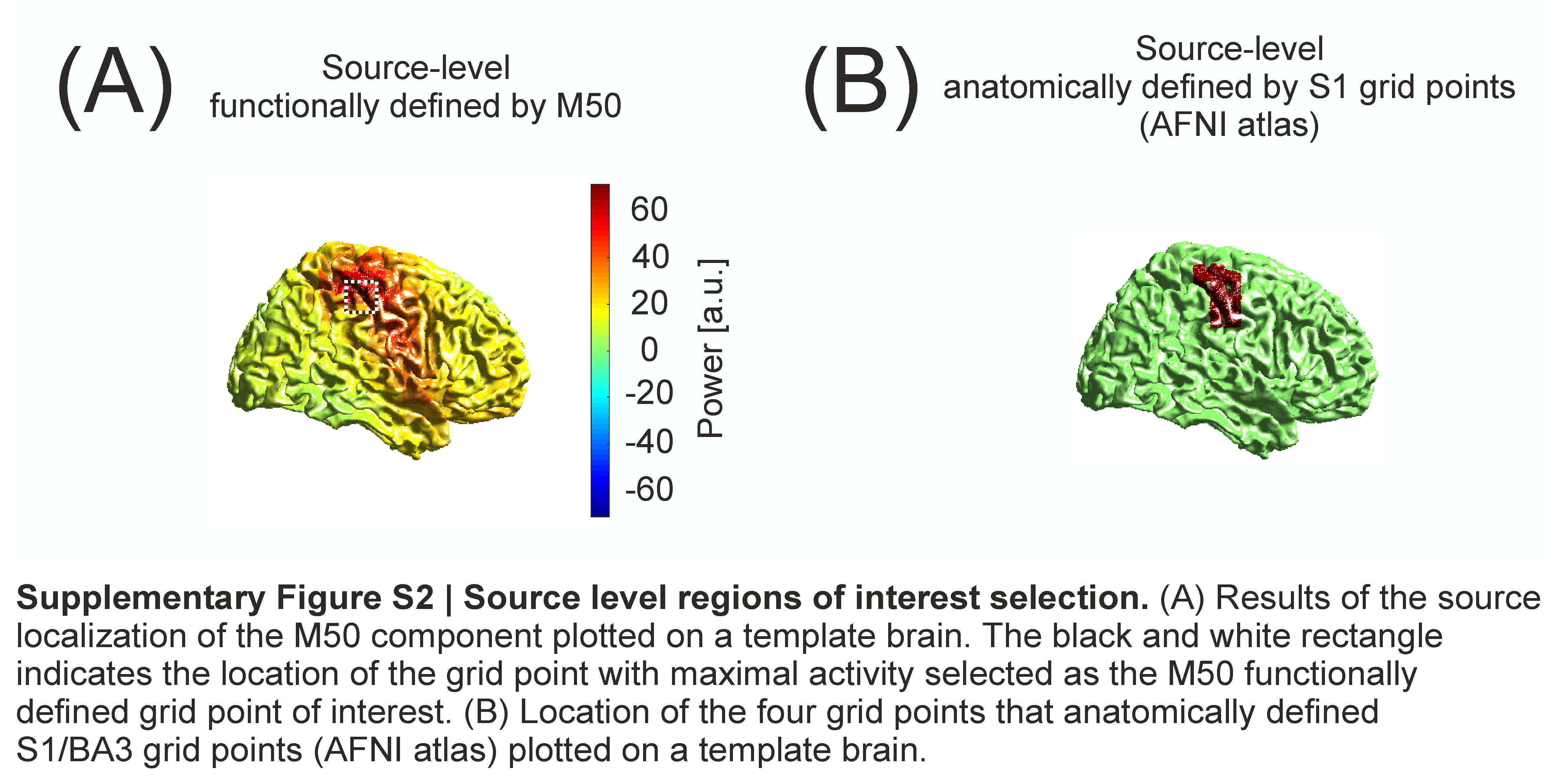

Supplement: Supplementary file 2 [file Image_2.tif]

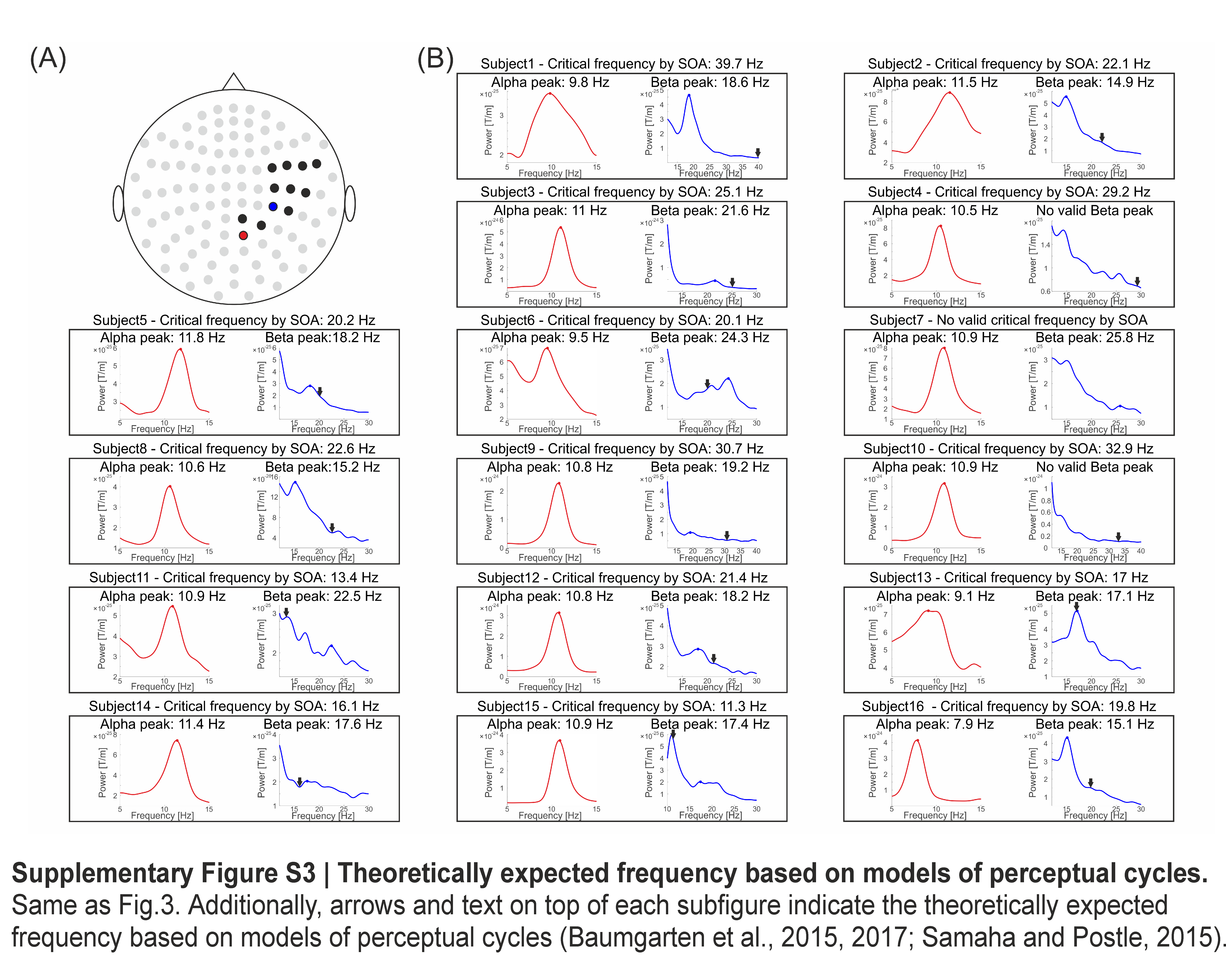

Supplement: Supplementary file 3 [file Image_3.tif]
